# Dengue virus 1 and 2: Epidemiology, host–virus interactions, clinical severity and integrated control strategies

**DOI:** 10.1016/j.parepi.2026.e00557

**Published:** 2026-10-10

**Authors:** Shairy Priya, Rishabha Malviya, Phool Chandra, Sathvik Belagodu Sridhar, Javedh Shareef, Tarun Wadhwa, Daniel Arockiam

**Affiliations:** a Department of Pharmacy, School of Medical and Allied Sciences, Galgotias University, Greater Noida, Uttar Pradesh, 201308, India; b Teerthanker Mahaveer College of Pharmacy, Teerthanker Mahaveer University, Moradabad, Uttar Pradesh, 244001, India; c College of Pharmacy, Dubai Medical University, Dubai, United Arab Emirates; d RAK College of Pharmacy, RAK Medical & Health Sciences University, Ras Al Khaimah, United Arab Emirates; e School of Engineering and IT, Manipal Academy of Higher Education, Dubai, United Arab Emirates; f Centre for Research Development, University of Cyberjaya, Persiaran Bestari, Cyber 11, Cyberjaya, Selangor, 63000, Malaysia

**Keywords:** DENV-1, DENV-2, Pathogenesis, Severity, Epidemiology, Control measures, Challenges

## Abstract

Dengue is a serious mosquito-borne viral disease caused by four antigenically distinct serotypes of the dengue virus, of which DENV-1 and DENV-2 have been found to play significant roles in outbreaks and severe disease across different geographical areas. This review discusses the epidemiology, host–virus interactions, pathogenesis, clinical manifestations, severity, vaccination, and integrated control strategies of DENV-1 and DENV-2. The relevant literature was identified by searching major scientific databases using keywords related to DENV-1, DENV-2, dengue epidemiology, pathogenesis, clinical severity, immune responses, vaccination, and vector control; studies were selected for relevance to the review's objectives. The evidence suggests that DENV-1 and DENV-2 share a number of major mechanisms in causing disease, including viral replication, innate immune modulation, endothelial dysfunction mediated by viral NS1, inflammatory response, and antibody-dependent enhancement, but that clinical outcomes are variable and may depend on viral genotype/lineage, prior dengue exposure, host immunity, age, geographical context, and study population. Furthermore, epidemiological and clinical studies have been unable to rank the severity of DENV-1 and DENV-2, as both serotypes have been reported to cause severe dengue across different settings and conditions. The available data thus suggest that the severity of dengue is multifactorial and not serotype-dependent. Ongoing comparative studies that integrate viral genetics, host immune responses, epidemiology, vaccines, and vector control are needed to enhance serotype-specific dengue prevention and clinical management.

## Graphical abstract

**Figure:**
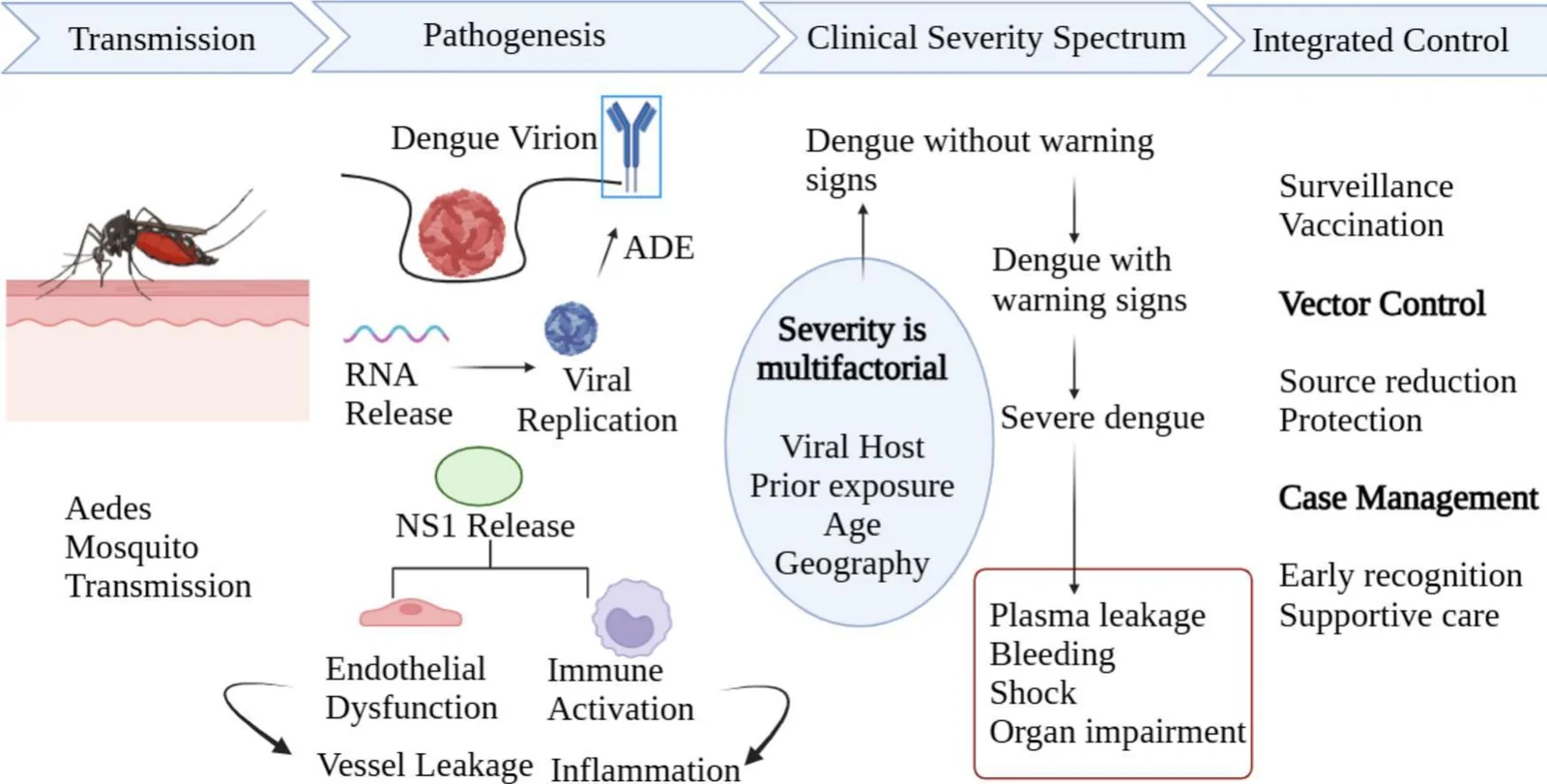

## 1 Introduction

Dengue is a vector-borne disease transmitted by mosquitoes to people. It is commonly found in tropical and subtropical regions. The symptoms of dengue include headache, high fever, body aches, rash, and nausea. Most symptomatic patients recover within 1–2 weeks. But in some cases, individuals often develop severe dengue, which can be fatal (*Dengue and Severe Dengue*, 2025). Worldwide, dengue spread is a major public threat. Its spread has increased about eightfold over the past 20 years. Approximately 3 billion people live in areas at risk of dengue transmission across more than 100 countries An estimated 100–400 million dengue virus infections occur annually worldwide, including infections that may not be detected through routine surveillance (*Dengue and Severe Dengue*, 2025; Nanaware et al., 2021).

Dengue virus (DENV) exists in four serotypes, namely DENV-1, DENV-2, DENV-3, and DENV-4. They have both genetic and antigenic distinctions. Survival from dengue type one infection prevents its reoccurrence, but does not protect from the other three types (Messina et al., 2014). Dengue virus is a positive-sense RNA, which is single-stranded, falls under the *Flaviviridae* family, and is primarily transmitted by vector agents, such as dengue virus is primarily transmitted by female *Aedes aegypti* and, to a lesser extent, *Aedes albopictus*, both of which can transmit multiple DENV serotypes (Kraemer et al., 2019). The increase in the spread of dengue is due to several factors, such as urbanization, migration or travel, and increasing population over the years (Kyle and Harris, 2008; Wilder-Smith et al., 2017). The crude death rate of DENV is not high, but it has subsequently impacted the hospitals and economic infrastructure of these nations (Stanaway et al., 2016; Yu et al., 2023). Globally, there are 96 million cases found annually, which are symptomatic (Bhatt et al., 2013).

According to the WHO 2009 classification, dengue is categorized as dengue without warning signs, dengue with warning signs, and severe dengue (Pan et al., 2019). The first stage of this disease is the acute febrile stage, during which the patient experiences symptoms such as nausea, aches, vomiting, and lethargy (WHO and TDR, 2009; Wang et al., 2020). The disease can show a self-limiting character, but only 2 to 5% of cases of dengue turn out to be severe, including platelet depletion and organ failure due to plasma leakage and hemorrhagic events. Management of severe dengue is primarily supportive and includes early recognition, careful fluid management, monitoring of hemodynamic status and organ function, and timely treatment of complications. Even though no confirmed therapeutics have been identified, *in vivo* and *ex vivo* studies show inhibition of dengue virus replication and virion formation (Perera et al., 2022; Kaptein et al., 2021; Yang et al., 2020; Goethals et al., 2023). Several efforts have been made to develop a vaccine, but due to DENV's diverse serotypes and the limited encouraging data and antibody-enhancing effects, it is quite challenging. However, the development of a new vaccine shows promising results when tested and has already received approval (Patel et al., 2023). Reported dengue cases are those detected through surveillance systems, whereas estimated infections include both detected and undetected cases. Symptomatic infections constitute only a proportion of total infections, while mortality refers specifically to deaths attributable to dengue. This review focuses specifically on DENV-1 and DENV-2, with emphasis on their epidemiology, comparative pathogenesis, host–virus interactions, clinical severity, immune responses, vaccination, and control strategies. Particular attention is given to distinguishing mechanisms shared by both serotypes from differences supported by comparative evidence. The review further evaluates the influence of viral genotype, host immunity, prior dengue exposure, and geographical context on disease severity, aiming to provide a balanced synthesis of current evidence and to identify key knowledge gaps.

A systematic literature review identified evidence on DENV-1 and DENV-2 epidemiology, pathogenesis, clinical severity, molecular evolution/structure, host–pathogen interactions, vaccination, and control. From 2000 to 2026, the PubMed/MEDLINE, Scopus, Web of Science, ScienceDirect, and Google Scholar databases were used to locate the publications. The search strategy used free-text terms for both serotypes (DENV-1, dengue virus serotype 1, and DENV-2, dengue virus serotype 2) AND for major domains of review (pathogenesis, epidemiology, clinical severity, vaccine, vector control, antibody-dependent enhancement, immune response, endothelial dysfunction, viral evolution). Boolean operators AND/OR were modified to the syntax used by each database. Peer-reviewed original studies, systematic reviews, meta-analyses, molecular epidemiological studies, surveillance reports, and relevant WHO/CDC reports on DENV-1 or DENV-2 were included. Conference abstracts, editorials, duplicate publications, non-English articles, and studies that lacked evidence of DENV-1/DENV-2 were excluded. The titles and abstracts were screened for relevance after duplicates were removed, and full texts were assessed for eligibility against the criteria. Each included study was characterized by its publication year, geographic location, study population, DENV serotype/−genotype, study design, epidemiological/clinical outcomes, major findings, and limitations. The evidence was then qualitatively synthesized, focusing on differences and similarities between DENV-1 and DENV-2. The last date of database search was 14 August 2026.

When findings were conflicting, greater emphasis was placed on studies with clearly defined diagnostic and clinical outcomes, appropriate comparative designs, larger or well-characterized study populations, and consistent serotype or genotype identification; differences in study population, geographical setting, viral genotype, previous dengue exposure, sampling period, and methodological design were considered when interpreting discrepant findings.The review was restricted to DENV-1 and DENV-2 to enable a focused comparative assessment of their epidemiology, molecular characteristics, host–virus interactions, clinical severity, immune responses, vaccination, and control strategies. These two serotypes were selected because sufficient epidemiological, clinical, molecular, and mechanistic evidence was available to support comparative synthesis across geographical settings. DENV-3 and DENV-4 were excluded because they were outside the predefined scope of this review; their exclusion does not imply lower epidemiological or clinical importance.

Epidemiological comparisons were made with preference for WHO and regional/national public health surveillance reports and complemented by peer-reviewed outbreak investigations/molecular epidemiological studies. The retrieved results comprised reported cases of dengue, serotype distribution, outbreak occurrence, hospitalization or severe dengue indicators (when available), mortality, and lineage or genotype replacement. Since the intensity of surveillance and the completeness of case reporting vary, the number of cases reported was not considered a direct reflection of the virulence of the specific serotype. Regional DENV-1 and DENV-2 impact scores were calculated by summarizing the consistency and relative predominance of serotype-specific evidence from the surveillance and epidemiological sources identified for each region for comparative visualization. The scores were standardized to enable comparisons between the two serotypes and were designed to measure relative epidemiological impact, not absolute prevalence or incidence. Limited or conflicting evidence in regions was interpreted with caution.

## 2 Comparative characteristics of dengue virus serotypes 1 and 2 (DENV-1 and DENV-2)

DENV-1 and DENV-2 are positive-sense single-stranded RNA virus strains of the Flavivirus genus of the Flaviviridae family, which are enveloped. They share a similar genomic structure containing seven non-structural proteins (regulatory), three membrane proteins, and three capsid proteins that are involved in viral replication, assembly, and immune evasion (Fig. 1) (Kuhn et al., 2002; Li et al., 2008; Guzman et al., 2010). They are transmitted primarily by *Aedes aegypti* and *Aedes albopictus* mosquitoes and confer long-lasting serotype-specific immunity, whereas cross-protection against heterologous serotypes is partial and temporary, leaving individuals vulnerable to secondary infection and antibody-dependent enhancement (ADE) (*Dengue | CDC Yellow Book* 2024, 2025; Leitmeyer et al., 1999). While these virological properties are more or less conserved, recent studies have indicated substantial differences in the epidemiology and clinical course of H5N1 infection, which are not explained solely by serotype.

**Fig. 1:**
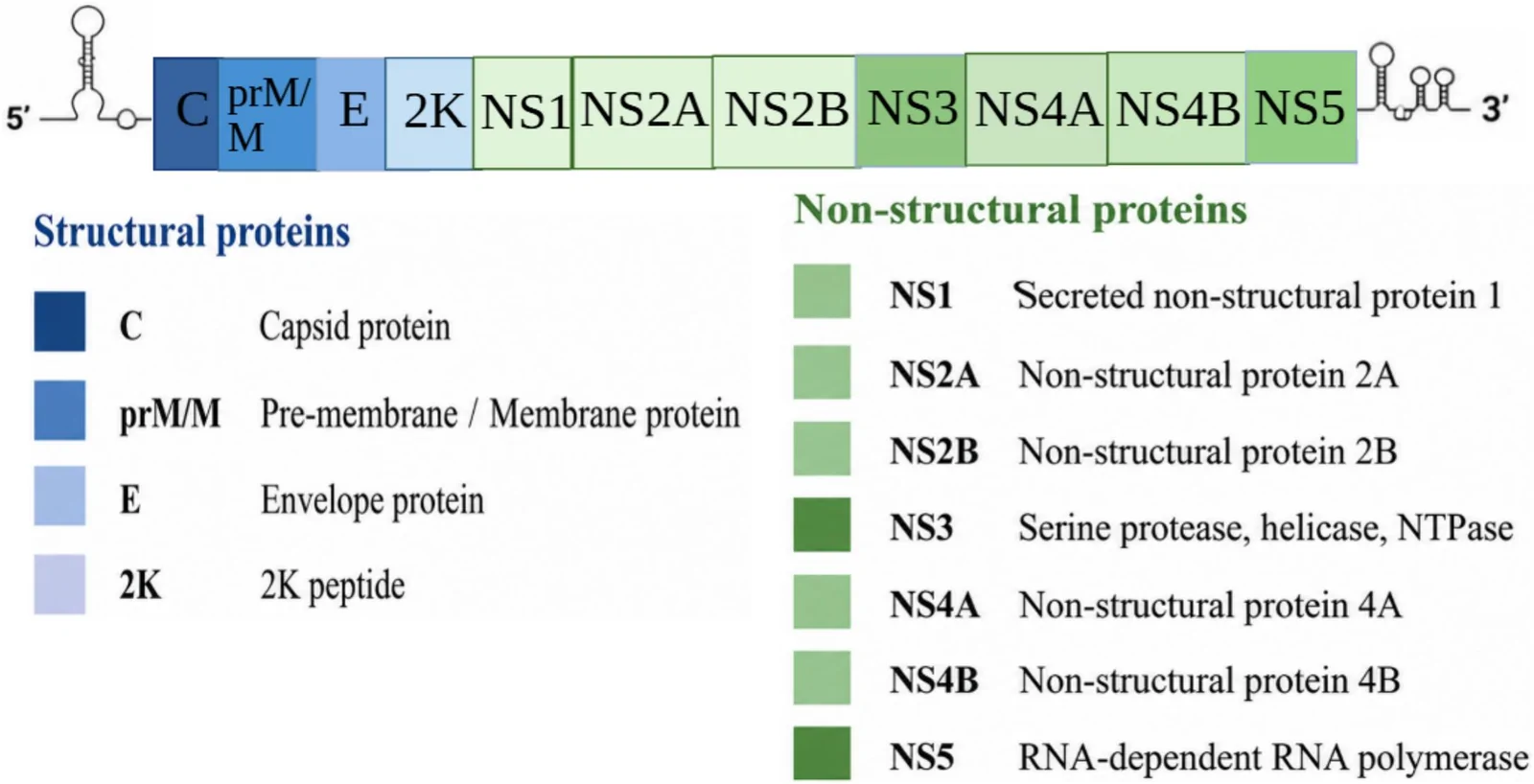
Genomic organization of the dengue virus genome.

Several epidemiological studies have reported higher frequencies of DHF and DSS with DENV-2 than DENV-1 in specific settings; however, this association is not consistent across all outbreaks. (Yung et al., 2015; Torres et al., 2021). However, subsequent outbreaks have demonstrated comparable severe disease during DENV-1 circulation, indicating that these historical associations do not establish an intrinsic serotype-specific hierarchy of virulence. (Yung et al., 2015; Torres et al., 2021). But in recent years, epidemiological evidence has called into question this traditional classification. DENV-1 can cause clinical severity equivalent to that of DENV-2, as evidenced by outbreaks in Singapore, Taiwan, and Nicaragua, whereas some DENV-2 epidemics had a predominantly mild clinical presentation. The results suggest that serotype is not a reliable indicator of disease severity; instead, viral genotype, host immunity, prior exposure, age, and geographic location are factors that affect severity (Ler et al., 2011; Balmaseda et al., 2006; Guzmán et al., 2000; Gupta et al., 2021). Such conflicting observations suggest that the severity of the disease is not solely determined by serotype and that the pathogenicity of the dengue viruses is critically dependent on other viral, host, and environmental factors. This variation is partly due to the large number of genetic differences between the two serotypes. DENV-1 has five major recognized genotypes (I to V), with I and V being the most common genotypes causing most recent outbreaks, and DENV-2 has five non-sylvatic recognized genotypes, which include the cosmopolitan genotype, which has a worldwide spread, and Asian lineage I and Asian lineage II, which are predominantly found in Asia. DENV-1 has five major recognized genotypes (I to V), of which I and V are the most common and have caused recent outbreaks; DENV-2 has five non-sylvatic recognized genotypes, including the Cosmopolitan genotype, which is widely distributed, and Asian lineage I and Asian lineage II, which are predominantly in Asia, as shown in Table 1. However, the distribution of genotypes is not always directly related to disease severity, and the same genotype has been associated with widely varying clinical patterns across endemic areas. These discrepancies indicate that it is inappropriate to consider viral evolution in isolation from host immunity, heterologous exposure history, vector competence, and regional transmission patterns.

**Table 1:** Study-level comparison of DENV-1 and DENV-2 severity.

Study	Serotype	Infection status	Population	Clinical outcome	Main finding	Interpretation/limitation	Reference
Singapore	DENV-1	Primary/secondary	Outbreak/clinical studies	Hospitalization/severe dengue	DENV-1 was associated with substantial disease burden during specific outbreaks	Severity was influenced by outbreak context and population immunity	(Ler et al., 2011; Balmaseda et al., 2006)
Taiwan	DENV-1	Primary/secondary	Epidemiological studies	Severe dengue/DHF	Severe disease was documented during DENV-1-dominant outbreaks	Does not establish intrinsic DENV-1 virulence	(Ler et al., 2011; Balmaseda et al., 2006; Guzmán et al., 2000; Gupta et al., 2021)
Nicaragua	DENV-1	Primary/secondary	Clinical/epidemiological studies	Severe dengue	DENV-1 circulation was associated with severe clinical outcomes	Host immune history and serotype sequence may contribute	(Ler et al., 2011; Balmaseda et al., 2006; Guzmán et al., 2000; Gupta et al., 2021)
Multiple epidemiological settings	DENV-2	Primary/secondary	Epidemiological studies	DHF/DSS/severe dengue	Higher frequencies of DHF/DSS have been reported with DENV-2 in some settings	Association is not consistent across geographical settings	(Yung et al., 2015; Torres et al., 2021)
Singapore	DENV-2	Primary/secondary	Outbreak studies	Severe dengue/hospitalization	DENV-2 has been associated with severe disease in some outbreaks	Findings vary with genotype, population immunity and outbreak context	(Yung et al., 2015; Ler et al., 2011)
Comparative studies	DENV-1 *vs* DENV-2	Primary/secondary	Clinical/epidemiological studies	Severe dengue/hospitalization	No serotype is consistently associated with greater severity across all populations	Viral genotype, viral load, age, immune history, host factors and geography modify outcomes	(Yung et al., 2015; Ler et al., 2011; Balmaseda et al., 2006; Guzmán et al., 2000; Gupta et al., 2021)

The increased pathogenicity often attributed to DENV-2 is also not fully understood. Experimental research has shown that DENV-2 can replicate more efficiently, cause more metabolic reprogramming of host glucose pathways, and elicit more inflammatory and endothelial responses than DENV-1 (Chutia et al., 2026; Sinha et al., 2024). The findings, however, have mostly been generated *in vitro* and in animal models, with some comparative clinical studies between DENV-1 and DENV-2 under standardized conditions. Thus, it is unclear whether the observed differences in virulence are biological or attributable to major factors such as antibody-dependent enhancement, host immune history, population genetics, and environmental factors.

All the available literature indicates, at best, a multifactorial model of dengue pathogenesis rather than a hierarchy of serotype virulence. Some epidemiological studies have documented that severe clinical symptoms are more common in the context of DENV-2 outbreaks, but there have been similar reports of severe cases occurring in a DENV-1-dominant outbreak. Hence, DENV-1 and DENV-2 are not necessarily mild or severe, as shown in Table 2. On the contrary, disease severity appears to stem from dynamic interactions among viral genotype, host immune responses, prior infection history, and epidemiological factors. The integrated multicenter studies involving sequencing of the viral genome, immunophenotyping, and standardized measures of clinical outcomes should thus be prioritized for future research to differentiate the effect of the different serotypes from the host- and environment-dependent determinants of severe dengue. This is key to better predicting outbreaks, better stratifying risk, and informing the development of next-generation vaccines and targeted therapeutics. Neurological involvement is an added dimension of severe dengue and can occur by multiple, but overlapping mechanisms. At present, three main pathways are supported by evidence: direct neuroinvasion, systemic complications of metabolic or hemodynamic disturbances, and post-infectious immune-mediated injury. Clinically, they are reported to be encephalitis, meningitis, myelitis, encephalopathy, Guillain–Barré syndrome, acute disseminated encephalomyelitis, and other neuromuscular complications. These heterogeneous manifestations suggest that neurological disease is not solely the result of direct viral replication in the nervous system; blood–brain barrier dysfunction, systemic inflammation, vascular abnormalities, and abnormal immune responses may also play a role. However, existing data are primarily based on case reports, small series, and mechanistic studies, thereby limiting quantitative comparisons between DENV-1 and DENV-2. Thus, neurological complications should be considered rare but not insignificant presentations of dengue, rather than a regular serotype-specific phenotype.

**Table 2:** Critical comparison of DENV-1 and DENV-2: evidence, controversies, and interpretation.

Parameter	DENV-1	DENV-2	Evidence supporting DENV-2 superiority	Conflicting evidence / limitations	Interpretation	Reference
Global epidemiology	Frequently predominates in Asia, the Americas, and the Western Pacific; responsible for several major epidemics (*Dengue | CDC Yellow Book* 2024, 2025; Yung et al., 2015; Patil et al., 2018).	Causes recurrent epidemics in Brazil, Southeast Asia, and Latin America (Torres et al., 2021; Martínez et al., 2024; Giovanetti et al., 2022).	Numerous surveillance studies associate DENV-2 with larger outbreaks and increased hospitalization rates.	Long-term surveillance demonstrates cyclical replacement between DENV-1 and DENV-2, indicating that epidemic dominance varies geographically and temporally rather than reflecting intrinsic transmissibility alone.	Outbreak magnitude is primarily determined by herd immunity, serotype replacement, vector abundance, and environmental conditions rather than serotype identity alone.	(*Dengue | CDC Yellow Book* 2024, 2025; Yung et al., 2015; Patil et al., 2018; Torres et al., 2021; Martínez et al., 2024; Giovanetti et al., 2022; *GenBank Overview*, 2025; *Dengue | Dengue*, 2025)
Genotype diversity	Five genotypes (I–V); Genotypes I and V predominate globally (Patil et al., 2018).	Five non-sylvatic genotypes; Cosmopolitan genotype shows the widest geographic distribution (Martínez et al., 2024; Giovanetti et al., 2022).	The Cosmopolitan genotype has been linked with rapid international dissemination and several recent outbreaks.	No genotype consistently produces more severe disease across different populations, and genotype–phenotype correlations remain inconsistent.	Viral evolution contributes to transmission dynamics, but genotype alone is an insufficient predictor of virulence.	(Patil et al., 2018; Martínez et al., 2024; Giovanetti et al., 2022; Bouzidi et al., 2026; Vaughn et al., 2000)
Clinical severity	Can produce the full clinical spectrum from mild dengue to severe dengue, including DHF and DSS.	Frequently associated with severe dengue, plasma leakage, DHF, and DSS in several settings, but severe outcomes also occur during DENV-1 outbreaks.	Several epidemiological studies report higher odds of severe disease during DENV-2 circulation; however, comparable severe outcomes have been documented during DENV-1-dominant outbreaks.	Severe DENV-1 outbreaks in Singapore, Taiwan, and Nicaragua reported hospitalization and mortality rates comparable to DENV-2 epidemics. Differences in study design and population immunity limit direct comparisons.	Clinical severity should be considered context-dependent, reflecting host–virus interactions rather than an intrinsic property of DENV-2.	(Yung et al., 2015; Torres et al., 2021; *Dengue | Dengue*, 2025; Endy et al., 2004; *DENGVAXIA*, 2025)
Viral replication and fitness	Efficient replication in susceptible cells.	Experimental studies demonstrate enhanced glucose metabolic reprogramming, higher replication efficiency, and greater viral fitness (Chutia et al., 2026; Sinha et al., 2024).	Laboratory studies consistently report increased replication efficiency and metabolic adaptation of DENV-2.	Most evidence originates from *in vitro* systems and animal models; prospective human validation is limited.	Enhanced replication may contribute to pathogenicity, but its independent clinical significance remains uncertain.	(Chutia et al., 2026; Sinha et al., 2024; Martina et al., 2009; Rodriguez-Roche and Gould, 2013)
Host immune interaction	Secondary heterologous infection promotes antibody-dependent enhancement (ADE) (*Dengue | CDC Yellow Book* 2024, 2025).	Similar dependence on ADE during secondary infection (Leitmeyer et al., 1999).	DENV-2 is more frequently identified in severe secondary infections.	ADE occurs irrespective of infecting serotype, and disease severity strongly depends on previous immune exposure and antibody titres.	Host immune history appears to exert a stronger influence on severe disease than serotype alone.	(*Dengue | CDC Yellow Book* 2024, 2025; Leitmeyer et al., 1999; *DENGVAXIA*, 2025; Chauhan et al., 2024)
Inflammatory response	Induces cytokine production and endothelial activation.	Frequently associated with elevated IL-6, TNF-α, IFN-γ, and increased vascular permeability.	Mechanistic studies suggest stronger inflammatory responses during DENV-2 infection.	Cytokine profiles differ substantially among studies because of variations in sampling time, disease stage, analytical methodology, and patient demographics.	Current evidence does not establish a unique inflammatory signature specific to DENV-2.	(Chutia et al., 2026; Sinha et al., 2024; Diamond and Pierson, 2015; Chen et al., 2018; Bhatt et al., 2020)
Evidence quality	Predominantly observational outbreak investigations and molecular epidemiology studies.	Supported by observational, experimental, and mechanistic investigations.	Larger volume of mechanistic evidence exists for DENV-2 than DENV-1.	Direct prospective head-to-head comparisons between DENV-1 and DENV-2 remain scarce, limiting causal inference.	The apparent superiority of DENV-2 partly reflects research intensity rather than unequivocal biological differences.	(*GenBank Overview*, 2025; *Dengue | Dengue*, 2025; *DENGVAXIA*, 2025; Pang et al., 2007)
Major knowledge gaps	Limited genotype-specific clinical cohorts and standardized severity assessments.	Limited multicenter genotype-specific validation despite extensive laboratory evidence.	Recent advances in genomics and metabolomics have improved understanding of DENV-2 biology.	Lack of standardized multicenter studies integrating virological, immunological, and clinical datasets prevents definitive conclusions regarding serotype-specific pathogenicity.	Future research should integrate viral genomics, immune profiling, and longitudinal clinical cohorts to distinguish intrinsic viral virulence from host-dependent determinants of severe dengue.	(*Dengue | Dengue*, 2025; *DENGVAXIA*, 2025; Pang et al., 2007; Halstead, 2019)

## 3 Comparative pathogenesis of DENV-1 and DENV-2

The basic steps of viral entry, genome replication, and virion assembly are highly conserved between DENV-1 and DENV-2 and are therefore unlikely to account for the marked differences in clinical outcomes reported among infected patients (Martina et al., 2009; Rodriguez-Roche and Gould, 2013; Chauhan et al., 2024; Diamond and Pierson, 2015; Chen et al., 2018; Bhatt et al., 2020; Pang et al., 2007; Halstead, 2019; Guabiraba and Ryffel, 2014). However, emerging data suggest that host immune response, endothelial dysfunction, and prior dengue exposure are more important than genotype-specific differences in viral replication in determining disease severity.

NS1 is the most important viral protein, as it directly compromises vascular integrity and stimulates the immune response, thus playing a key role in dengue pathogenesis. Secreted NS1 binds to Toll-like receptor 4 (TLR4) and activates inflammatory signaling pathways, which induce the release of tumor necrosis factor alpha (TNF-α), interleukin 6 (IL-6), and IL-1 beta (IL-1β); it also disrupts the endothelial glycocalyx by activating heparanase, which enhances vascular permeability and plasma leakage (Chauhan et al., 2024; Diamond and Pierson, 2015; Chen et al., 2018; Bhatt et al., 2020). Current evidence, however, does not conclusively show that NS1 of a single serotype causes more endothelial injury than that of the other, and that these mechanisms are shared by both DENV serotypes. Without standardized comparative studies, firm statements about serotype-specific NS1 pathogenicity are not possible.

DENV-2 is often thought to be more pathogenic, but the evidence is less consistent. It has been reported in experimental studies that DENV-2 infection leads to improved metabolic reprogramming, increased activation of the NLRP3 inflammasome and production of IL-1β, as well as endothelial dysfunction (Chutia et al., 2026; Sinha et al., 2024; Shrivastava et al., 2020). Observations are consistent with plausible mechanistic explanations for increases in severe dengue in some epidemiological studies. However, the majority of results reported here have been obtained from cell culture and animal studies, and head-to-head clinical comparisons between DENV-1 and DENV-2 are still limited. However, similar disease severity has been reported in several DENV-1-dominated outbreaks, suggesting that this is not solely due to DENV-2, as shown in Table 3.

**Table 3:** Critical comparison of pathogenic mechanisms between DENV-1 and DENV-2.

Pathogenic feature	DENV-1	DENV-2	Evidence supporting DENV-2 pathogenicity	Conflicting evidence / limitations	Critical interpretation	References
Viral replication	Efficient replication following receptor-mediated entry and RNA translation.	Experimental studies indicate enhanced replication efficiency and metabolic reprogramming.	Increased glucose metabolism and viral fitness have been reported in experimental models.	Most evidence is derived from *in vitro* studies; direct comparative clinical validation remains limited.	Replication efficiency alone does not adequately explain differences in disease severity.	(Chutia et al., 2026; Sinha et al., 2024; Rodriguez-Roche and Gould, 2013; Diamond and Pierson, 2015; Guabiraba and Ryffel, 2014)
NS1-mediated endothelial injury	NS1 activates TLR4 signaling and disrupts the endothelial glycocalyx, promoting plasma leakage.	Similar mechanism with reports of greater endothelial dysfunction during severe DENV-2 infection.	Mechanistic studies associate DENV-2 NS1 with increased vascular permeability.	Direct comparisons of NS1 activity between DENV-1 and DENV-2 are scarce.	NS1 is a major virulence factor, but serotype-specific differences remain incompletely resolved.	(Chauhan et al., 2024; Chen et al., 2018; Bhatt et al., 2020; Shrivastava et al., 2020)
Inflammatory response	Induces TNF-α, IL-6, IL-8 and IL-10, contributing to endothelial activation.	Frequently associated with stronger NLRP3 inflammasome activation and increased IL-1β release.	Experimental studies suggest greater inflammatory activation during DENV-2 infection.	Cytokine profiles vary according to sampling time, disease stage and patient population.	Current evidence does not identify a unique inflammatory signature exclusive to DENV-2.	(Chen et al., 2018; Pang et al., 2007; Shrivastava et al., 2020)
Immune evasion	NS5 suppresses type-I interferon signaling to facilitate viral persistence.	Similar interferon antagonism with reports of enhanced immune modulation.	Laboratory studies indicate more efficient immune suppression in DENV-2.	Evidence is predominantly experimental rather than clinical.	Immune evasion is conserved among serotypes, and quantitative differences remain uncertain.	(Diamond and Pierson, 2015; Halstead, 2019; Guabiraba and Ryffel, 2014)
Antibody-dependent enhancement (ADE)	Secondary heterologous infection facilitates Fcγ receptor-mediated viral entry and enhanced replication.	Identical mechanism contributes to severe secondary dengue.	DENV-2 is frequently isolated during severe secondary infections.	ADE depends primarily on previous immune exposure rather than infecting serotype.	Host immune history is likely a stronger determinant of severe dengue than serotype identity alone.	(Martina et al., 2009; Pang et al., 2007)
Endothelial dysfunction	Plasma leakage results from NS1 activity and inflammatory cytokines.	Often associated with greater vascular permeability during severe disease.	Clinical studies frequently report more severe plasma leakage during DENV-2 outbreaks.	Comparable endothelial injury has also been observed during major DENV-1 epidemics.	Endothelial dysfunction reflects combined viral and host factors rather than serotype alone.	(Chen et al., 2018; Bhatt et al., 2020; Pang et al., 2007; Shrivastava et al., 2020)
Clinical implications	Can cause classical dengue, DHF and DSS.	More frequently linked with severe dengue, hospitalization and DSS.	Multiple epidemiological investigations associate DENV-2 with severe outbreaks.	Several DENV-1 outbreaks have demonstrated comparable severity, questioning a universal virulence hierarchy.	Clinical outcome should be interpreted within the context of host immunity, viral genotype and epidemiological setting rather than serotype alone.	(Chutia et al., 2026; Sinha et al., 2024; Martina et al., 2009; Chen et al., 2018; Bhatt et al., 2020; Pang et al., 2007; Halstead, 2019; Guabiraba and Ryffel, 2014; Shrivastava et al., 2020)
Overall evidence quality	Evidence largely derived from observational studies and molecular investigations.	Supported by observational, mechanistic and experimental studies.	Greater volume of mechanistic literature exists for DENV-2.	Direct prospective head-to-head comparisons remain limited.	The apparent superiority of DENV-2 partly reflects greater research emphasis rather than unequivocal biological differences.	(Chutia et al., 2026; Sinha et al., 2024; Martina et al., 2009; Rodriguez-Roche and Gould, 2013; Chauhan et al., 2024; Diamond and Pierson, 2015; Chen et al., 2018; Bhatt et al., 2020; Pang et al., 2007; Halstead, 2019; Guabiraba and Ryffel, 2014; Shrivastava et al., 2020)

Another factor is antibody-dependent enhancement (ADE), a biological mechanism in which pre-existing sub-neutralizing antibodies facilitate Fcγ-receptor-mediated viral uptake into immune cells, potentially enhancing viral replication and cytokine production (Martina et al., 2009; Pang et al., 2007). Importantly, ADE is not serotype-specific and is primarily dependent on the order of heterologous infections and the host immune landscape. Therefore, host immune history tends to play a greater role than viral serotype in determining clinical severity, which is why clinical responses can vary greatly among patients infected with the same serotype.

Overall, the available evidence favors a multifactorial rather than serotype-dependent model of dengue pathogenesis. Based on the available literature, disease progression depends on a combination of viral genotype, host genetics, immune status, prior dengue exposure, and regional epidemiological factors. The available literature suggests that disease progression is determined by a combination of viral genotype, host genetics, immune status, disease history, and epidemiology. Therefore, future multicentre investigations will need to combine viral genomics, longitudinal immunological characterization, and clinical standardized phenotyping to differentiate between serotype-specific pathogenic mechanisms and host-mediated determinants of severe dengue.

## 4 Molecular determinants of disease severity and viral evolution

There is current evidence that the clinical severity of DENV-1 and DENV-2 depends not only on serotype but also on complex interactions among viral evolution, host immune responses, and epidemiological factors. While the genomic organization and the replication strategy are highly conserved, ongoing viral evolution generates genetically distinct lineages that differ in their transmission potential and epidemic potential. However, the few studies conducted have not consistently shown that any given genotype is more virulent than others; thus, viral evolution is more likely to play a role in outbreak dynamics than in severity. Therefore, the serotype pathogenicity needs to be interpreted taking into account the genetic variation of the virus and host factors (Patil et al., 2018; Martínez et al., 2024; Giovanetti et al., 2022; Ler et al., 2011; Anwar et al., 2020; Ma et al., 2021; Jiang et al., 2022).

The epidemiology of dengue is further complicated by the presence of genotype diversity and lineage replacement. Both DENV-1 and DENV-2 consist of several circulating genotypes with shifting geographical distributions over time. Multiple large outbreaks have occurred in conjunction with the emergence of novel lineages and the replacement of previously dominant lineages, such as in Singapore, Taiwan, Brazil, China, and India (Ler et al., 2011; Anwar et al., 2020; Ma et al., 2021; Jiang et al., 2022). However, similar clinical outcomes have been observed in outbreaks involving different genotypes, indicating that lineage replacement is not the sole explanation for increased clinical severity. However, more likely that interactions among viral adaptation, waning herd immunity, vector competence, and environmental pressures drive the epidemic expansion of the virus, rather than intrinsic virulence.

The connection between viral fitness and pathogenicity is also not fully understood. Experimental studies have shown that DENV-2 may exhibit increased replication capacity, metabolic reprogramming of host glucose pathways, and greater activation of inflammatory signaling compared with some DENV-1 isolates (Chutia et al., 2026; Sinha et al., 2024). These results offer plausible mechanisms for the increased reports of severe dengue observed during many DENV-2 outbreaks. Most of the supporting evidence comes from *in vitro* studies or animal experiments; however, severe epidemics of DENV-1 have been reported with hospitalization and mortality rates that are similar to those reported during the epidemic of DENV-2 (Ler et al., 2011; Balmaseda et al., 2006). This discrepancy suggests that the virus's fitness in the laboratory is not always correlated with its pathogenicity in humans.

The mechanisms of host–pathogen interactions remain key to understanding the pathogenesis of dengue. Viral infection triggers an innate immune response by dendritic cells, monocytes, and macrophages, leading to the secretion of type I interferons and inflammatory mediators. Dengue virus also uses non-structural proteins to downregulate interferon signaling and escape the immune system, thereby establishing productive infections (Diamond and Pierson, 2015; Halstead, 2019; Guabiraba and Ryffel, 2014). Of these proteins, secreted NS1 has been identified as a major contributor to vascular injury through its activation of TLR4-dependent inflammatory pathways, its capacity to activate the complement pathway, and its ability to degrade the endothelial glycocalyx and increase vascular permeability (Chauhan et al., 2024; Diamond and Pierson, 2015; Chen et al., 2018; Bhatt et al., 2020; Shrivastava et al., 2020). While these mechanisms are well supported by experiments, direct comparison of DENV-1- and DENV-2-derived NS1 proteins remains limited, and definitive conclusions about the differences in endothelial injury associated with the different serotypes cannot yet be drawn.

Dysregulated immune activation is also a defining feature of severe dengue. Elevated levels of IL-6, IL-8, IL-10, TNF-α, IFN-γ, and other inflammatory mediators have been associated with endothelial dysfunction and plasma leak (Chen et al., 2018; Pang et al., 2007; Shrivastava et al., 2020). However, cytokine profiles have shown significant differences across studies due to disease stage, sampling time, patient characteristics, and analytical techniques, and therefore cannot serve as a cross-study marker of severe disease. Similarly, antibody-dependent enhancement (ADE) has been one of the most widely accepted explanations for severe secondary infection, while virus-specific T-cell responses are responsible for viral clearance and, at times, immunopathology. These observations suggest that the disease's severity is due to a complex interaction of viral characteristics, innate immunity, adaptive immune response, and host susceptibility, as cytokine dysregulation was not observed to be the sole cause of severe dengue (Martina et al., 2009; Pang et al., 2007).

The virus-induced metabolic reprogramming is also tightly associated with the pathogenesis of Dengue and could help link cellular energy metabolism to immune dysregulation. DENV infection can induce a glycolytic switch, influence lipid metabolism (lipid droplet utilization, lipophagy), and modulate amino acid metabolism for viral replication and biosynthetic needs. Mitochondrial dynamics disruption may also exacerbate the generation of reactive oxygen species, thereby contributing to inflammatory signaling and the antiviral response. These modifications suggest that metabolic reprogramming is not simply the result of viral replication but can be a key determinant of the extent and duration of host immune activation. Importantly, no consistent DENV-1 *versus* DENV-2 metabolic signature has been identified to date, and metabolic alterations are considered a shared host–virus process; the role of this process in the severity of diseases caused by the two serotypes should be validated in future clinical studies.

Taken together, the literature reviewed in this study calls into question the widely held view that DENV-2 is more pathogenic than DENV-1. On the contrary, clinical severity seems to be determined more by the dynamic interplay among viral evolution, genotype diversity, genotype replacement, immune escape, endothelial dysfunction, and host immune history than by a mere serotype. One of the current challenges for the field is that most of the available evidence derives from observational outbreak investigations and experimental models, while prospective multicentre studies that combine viral genomics, immunophenotyping, transcriptomics, and standardization of clinical outcomes remain limited. These knowledge gaps will be crucial to the identification of reliable molecular markers of severe dengue, the development of next-generation vaccines and targeted antiviral therapies, and improved outbreak forecasting, as shown in Table 4 (*Dengue | Dengue*, 2025; *DENGVAXIA*, 2025; Pang et al., 2007; Halstead, 2019). The available evidence does not justify classifying either DENV-1 or DENV-2 as universally mild or severe. The clinical outcome depends on the virus genotype, the sequence of infection (primary or secondary), the host immune system, age, geographical setting, and the studied population.

**Table 4:** Critical comparison of DENV-1 and DENV-2: current evidence, controversies, and research gaps.

Comparative parameter	DENV-1	DENV-2	Evidence supporting DENV-2 superiority	Conflicting evidence / limitations	Critical interpretation	Reference
Global circulation	Predominant in several outbreaks across Asia, Taiwan, Singapore and the Americas.	Frequently responsible for recurrent epidemics in Brazil, Southeast Asia and Latin America.	Higher frequency of severe outbreaks has often been reported during DENV-2 circulation.	Dominant serotype varies geographically and temporally owing to herd immunity and serotype replacement.	Epidemic dominance reflects ecological and immunological pressures rather than intrinsic viral superiority.	(Patil et al., 2018; Torres et al., 2021; Martínez et al., 2024; Giovanetti et al., 2022; Ler et al., 2011; Anwar et al., 2020; Ma et al., 2021; Jiang et al., 2022)
Genotype diversity	Five genotypes (I–V); Genotypes I and V commonly associated with outbreaks.	Five non-sylvatic genotypes; Cosmopolitan genotype is the most widespread.	Cosmopolitan genotype has shown efficient international dissemination.	No genotype consistently produces greater clinical severity across populations.	Genotype influences transmission but is an unreliable predictor of virulence.	(Patil et al., 2018; Martínez et al., 2024; Giovanetti et al., 2022; Ma et al., 2021; Jiang et al., 2022)
Lineage replacement	Replacement by genotype III preceded major Singapore and Taiwan outbreaks.	Frequent lineage shifts reported in Brazil and China.	Emergence of new DENV-2 lineages has coincided with several epidemics.	Temporal association does not establish causality; host immunity may drive lineage replacement.	Integrated genomic and epidemiological surveillance is required to distinguish adaptation from stochastic replacement.	(Ler et al., 2011; Anwar et al., 2020; Ma et al., 2021; Jiang et al., 2022)
Replication and viral fitness	Efficient intracellular replication.	Enhanced glucose metabolic reprogramming and replication efficiency reported experimentally.	Laboratory studies indicate greater replication kinetics and metabolic adaptation of DENV-2.	Evidence is largely derived from cell culture and animal models; clinical validation remains limited.	Experimental viral fitness cannot be directly equated with increased human pathogenicity.	(Chutia et al., 2026; Sinha et al., 2024; Halstead, 2019; Guabiraba and Ryffel, 2014)
Immune escape	NS proteins inhibit interferon signaling.	Similar immune evasion mechanisms through NS proteins.	Greater suppression of innate immunity has been proposed for DENV-2.	Direct head-to-head comparisons between serotypes are scarce.	Immune escape appears to be a conserved flaviviral strategy rather than a serotype-specific property.	(Diamond and Pierson, 2015; Halstead, 2019; Guabiraba and Ryffel, 2014)
NS1-mediated vascular leakage	NS1 activates TLR4 and disrupts endothelial glycocalyx.	Similar mechanism with strong association with severe dengue.	Experimental evidence consistently links NS1 with endothelial dysfunction and plasma leakage.	Comparative functional studies of DENV-1 and DENV-2 NS1 remain limited.	Host immune responses likely modulate NS1 pathogenicity more strongly than serotype identity.	(Chauhan et al., 2024; Diamond and Pierson, 2015; Chen et al., 2018; Bhatt et al., 2020; Shrivastava et al., 2020)
Inflammatory response	Elevated IL-6, IL-8, IL-10 and TNF-α during severe disease.	Frequently associated with stronger cytokine responses and vascular permeability.	Several mechanistic studies report greater inflammatory activation during DENV-2 infection.	Cytokine profiles differ according to disease stage, sampling time and patient characteristics.	No unique cytokine signature reliably differentiates severe DENV-1 from severe DENV-2 infection.	(Chen et al., 2018; Pang et al., 2007; Shrivastava et al., 2020)
Clinical severity	Can produce classical dengue, DHF and severe dengue.	More frequently associated with DHF, DSS and hospitalization.	Multiple epidemiological studies associate DENV-2 with severe clinical outcomes.	Large DENV-1 outbreaks have demonstrated comparable hospitalization and mortality rates.	Disease severity is context-dependent and reflects virus–host interactions rather than serotype alone.	(Fried et al., 2010; Nisalak et al., 2003; Yacoub and E. M. W. W. W. W, 2017; Chinnawirotpisan et al., 2008; Ler et al., 2011; Balmaseda et al., 2006; Guzmán et al., 2000; Gupta et al., 2021)
Evidence quality	Predominantly outbreak investigations and observational studies.	Greater number of experimental and mechanistic studies available.	Larger body of laboratory evidence exists for DENV-2.	Few prospective multicentre comparative studies directly evaluate DENV-1 and DENV-2.	The apparent superiority of DENV-2 may partly reflect research intensity rather than biological differences.	(Chutia et al., 2026; Sinha et al., 2024; Fried et al., 2010; Nisalak et al., 2003; Yacoub and E. M. W. W. W. W, 2017; Chinnawirotpisan et al., 2008; Ler et al., 2011; Balmaseda et al., 2006; Guzmán et al., 2000; Gupta et al., 2021)
Knowledge gaps	Limited genotype-specific clinical studies.	Limited multicentre validation of genotype-dependent pathogenicity.	Advances in genomics and immunology have improved mechanistic understanding.	Lack of integrated genomic, immunological and longitudinal clinical datasets prevents definitive conclusions.	Future studies should combine viral genomics, immune profiling and standardized clinical phenotyping to identify reliable predictors of severe dengue.	(*Dengue | Dengue*, 2025; *DENGVAXIA*, 2025; Pang et al., 2007; Halstead, 2019)

## 5 Comparative clinical severity of DENV-1 and DENV-2

Dengue is no more virulent, as observed from the increasing burden of dengue in the world. While there has been a rise from 505,430 cases in 2000 to >5.2 million cases in 2019, and modeling studies estimate that around 390 million people are infected with Aedes vector-borne serotype pathogens annually, with only 96 million being clinically apparent, this is mainly due to increased distribution of Aedes vectors, climate change, urbanization, enhanced surveillance, and greater mobility between countries (Bouzidi et al., 2026; Guha-Sapir and Schimmer, 2005; Waggoner et al., 2016; Brady et al., 2012). So, the serotype-specific disease severity should not be interpreted as an indicator of epidemiological expansion.

Whether DENV-2 is intrinsically more virulent than DENV-1 remains unresolved. Many epidemiological studies have correlated DENV-2 with increased incidence of dengue hemorrhagic fever (DHF), dengue shock syndrome (DSS), thrombocytopenia, plasma leakage, and hospitalization of children, but severe outbreaks have also been associated with DENV-1 predominance. The observations imply that serotype is not a good indicator of disease intensity. Many epidemiological studies have correlated DENV-2 with increased dengue hemorrhagic fever (DHF), dengue shock syndrome (DSS), thrombocytopenia, plasma leakage, and pediatric hospitalization (Fried et al., 2010; Nisalak et al., 2003; Yacoub and E. M. W. W. W. W, 2017; Chinnawirotpisan et al., 2008). Based on these observations, DENV-2 is considered to be the most virulent serotype. But several well-documented outbreaks of DENV-1 have occurred, which contradict this. For instance, the 2005 Singapore epidemic (with a higher incidence of DENV-1) had an incidence rate of 326.5/100,000 compared with 193.3/100,000 of the subsequent DENV-2 outbreak in 2007 (Ler et al., 2011). Likewise, multivariate analyses adjusted for age, sex, recruitment site, year of infection, fever duration, and infection status were conducted, and DENV-1 was independently significantly associated with severe dengue and DHF (Balmaseda et al., 2006). The results suggest that outbreaks cannot be reliably classified as mild or severe, or as having a good or bad prognosis, based on the serotype.

The conflicting results reported in different studies are probably because of the diversity in viral genotype, host immunity, antibody-dependent enhancement (ADE), viral load, demographic factors, and regional transmission dynamics between the two viruses, DENV-1 and DENV-2, and not because of any intrinsic differences in the biology of the two viruses (Sangkawibha et al., 1984; Rico-Hesse et al., 1997; Guzman and Kouri, 2003; Burke et al., 1988). This conclusion is also supported by clinical observations that DENV-1 infection is associated with elevated viral RNA levels and increased ocular manifestations, whereas DENV-2 infection is associated with arthralgia and thrombocytopenia (Balmaseda et al., 2006). In contrast, another cohort study of 100 laboratory-confirmed dengue cases found that the most prevalent circulating serotype (34%) was DENV-2 and that it was most commonly associated with severe disease (Gupta et al., 2021). These studies do not define a virulence hierarchy but collectively show that the clinical phenotype may vary with the outbreak context and interactions between the host and the virus.

Another constraint is that most of the comparative evidence comes from retrospective observational outbreak studies, which vary widely in patient inclusion criteria, circulating genotypes, diagnostic criteria, and severity categories. The methodological differences make it hard to compare findings across studies and can, at least in part, account for conflicting results regarding the virulence of each serotype. Based on the existing evidence, this suggests that severe Dengue is not due to a more virulent DENV-2 strain but rather to a context-dependent disease, as shown in Table 5. To distinguish true viral serotype-specific pathogenicity from host-dependent factors in disease severity, future multicentre prospective studies combining viral genomics, quantitative viral load measurements, and standardized clinical phenotyping and longitudinal immunological profiling are needed.

**Table 5:** Critical appraisal of the comparative clinical severity of DENV-1 and DENV-2.

Clinical parameter	Evidence associating DENV-1 with the outcome	Evidence associating DENV-2 with the outcome	Conflicting evidence	Critical interpretation	Knowledge gap	References
Global outbreak burden	DENV-1 has been associated with major outbreaks, including the Singapore 2005 outbreak (326.5 cases/100,000 population) (Ler et al., 2011).	DENV-2 has frequently predominated during outbreaks in Latin America and Southeast Asia and has been associated with recurrent epidemics (Fried et al., 2010; Nisalak et al., 2003; Yacoub and E. M. W. W. W. W, 2017; Chinnawirotpisan et al., 2008).	Epidemic magnitude varies substantially by region and year because of herd immunity, vector abundance, serotype replacement and other epidemiological factors.	Outbreak magnitude reflects transmission dynamics and population susceptibility rather than intrinsic serotype virulence.	Quantitative multicountry comparisons controlling for transmission intensity remain limited.	(Fried et al., 2010; Nisalak et al., 2003; Yacoub and E. M. W. W. W. W, 2017; Chinnawirotpisan et al., 2008; Ler et al., 2011; Balmaseda et al., 2006)
Risk of severe dengue, DHF and DSS	Multivariate analyses have identified DENV-1 as an independent predictor of severe dengue and DHF after adjustment for selected demographic and clinical variables (Balmaseda et al., 2006).	Several epidemiological studies have reported associations between DENV-2 and increased DHF, DSS and hospitalization, particularly in children (Fried et al., 2010; Nisalak et al., 2003; Yacoub and E. M. W. W. W. W, 2017; Chinnawirotpisan et al., 2008).	Findings differ according to geographical setting, infection history, population characteristics and study design.	Current evidence does not establish a universal severity hierarchy between DENV-1 and DENV-2.	Large prospective comparative cohorts with standardized severity definitions are limited.	(Fried et al., 2010; Nisalak et al., 2003; Yacoub and E. M. W. W. W. W, 2017; Chinnawirotpisan et al., 2008; Ler et al., 2011; Balmaseda et al., 2006; Gupta et al., 2021)
Clinical manifestations	Higher viral RNA levels and increased ocular involvement have been reported in some DENV-1 studies (Balmaseda et al., 2006).	DENV-2 has been associated with thrombocytopenia, arthralgia and severe plasma leakage in several studies (Fried et al., 2010; Nisalak et al., 2003; Yacoub and E. M. W. W. W. W, 2017; Chinnawirotpisan et al., 2008; Balmaseda et al., 2006).	Clinical phenotypes vary substantially across regions and patient populations.	Clinical manifestations are influenced by viral and host determinants and should not be attributed to serotype alone.	Standardized prospective comparisons of clinical phenotypes are needed.	(Fried et al., 2010; Nisalak et al., 2003; Yacoub and E. M. W. W. W. W, 2017; Chinnawirotpisan et al., 2008; Ler et al., 2011; Balmaseda et al., 2006)
Hospitalization and disease severity	Severe disease has been documented during DENV-1 outbreaks, indicating that DENV-1 should not be considered uniformly less severe than DENV-2 (Ler et al., 2011; Balmaseda et al., 2006).	DENV-2 has been associated with higher hospitalization or intensive-care admission rates in some epidemiological studies (Fried et al., 2010; Nisalak et al., 2003; Yacoub and E. M. W. W. W. W, 2017; Chinnawirotpisan et al., 2008).	Hospitalization rates are influenced by healthcare access, surveillance practices, admission criteria and outbreak characteristics.	Hospitalization is not a direct surrogate for intrinsic viral virulence.	Harmonized severity and hospitalization criteria across comparative studies are needed.	(Fried et al., 2010; Nisalak et al., 2003; Yacoub and E. M. W. W. W. W, 2017; Chinnawirotpisan et al., 2008; Ler et al., 2011; Balmaseda et al., 2006)
Determinants of severe disease	Secondary heterologous infection, high viral load and host immune status have been associated with severe disease in DENV-1 infection (Sangkawibha et al., 1984; Rico-Hesse et al., 1997; Guzman and Kouri, 2003; Burke et al., 1988).	Similar determinants have been reported for DENV-2 infection (Sangkawibha et al., 1984; Rico-Hesse et al., 1997; Guzman and Kouri, 2003; Burke et al., 1988).	These determinants operate across dengue serotypes and cannot independently explain serotype-specific severity.	Host immune history, viral genotype/lineage and viral burden may substantially influence clinical outcome.	Integrated genomic, virological and immunological studies remain limited.	(Sangkawibha et al., 1984; Rico-Hesse et al., 1997; Guzman and Kouri, 2003; Burke et al., 1988)
Quality of available evidence	Evidence for DENV-1 severity is derived largely from observational and outbreak investigations, with heterogeneous study populations and methodologies.	DENV-2 has been investigated extensively in epidemiological and mechanistic studies, but direct comparative evidence remains limited.	Direct head-to-head prospective comparisons are uncommon and methodological heterogeneity limits cross-study interpretation.	Differences between serotypes should therefore be interpreted as study-specific associations rather than definitive evidence of intrinsic virulence differences.	Well-designed multicentre prospective comparative studies are required.	(Sangkawibha et al., 1984; Rico-Hesse et al., 1997; Guzman and Kouri, 2003; Burke et al., 1988; Fried et al., 2010; Nisalak et al., 2003; Yacoub and E. M. W. W. W. W, 2017; Chinnawirotpisan et al., 2008; Ler et al., 2011; Balmaseda et al., 2006; Guzmán et al., 2000; Gupta et al., 2021)
Overall conclusion	DENV-1 can cause substantial outbreaks and severe dengue under specific epidemiological and host-related conditions.	DENV-2 has been associated with severe clinical outcomes in several studies, although this association is not consistent across all populations and settings.	Existing evidence does not provide unanimous evidence that either serotype is intrinsically more virulent.	Current evidence supports a context-dependent model of dengue severity, involving serotype, genotype, infection sequence, host immunity, viral burden and geographical context.	Future studies should integrate viral genomics, quantitative viral load, immune profiling and standardized clinical phenotyping.	(Sangkawibha et al., 1984; Rico-Hesse et al., 1997; Guzman and Kouri, 2003; Burke et al., 1988; Fried et al., 2010; Nisalak et al., 2003; Yacoub and E. M. W. W. W. W, 2017; Chinnawirotpisan et al., 2008; Ler et al., 2011; Balmaseda et al., 2006; Guzmán et al., 2000; Gupta et al., 2021)

## 6 Global epidemiology and serotype dynamics

Dengue is expanding globally, but not due to the development of a more virulent serotype. The disease burden from Dengue is nearly 70% in Asia, and currently the disease is endemic in more than 100 countries (Bouzidi et al., 2026). In 2023, more than 4.5 million cases and about 2300 deaths were reported in the Region of the Americas by WHO, and significant outbreaks occurred in Vietnam (396,000 cases, 4 deaths), Bangladesh (321,000 cases, 6 deaths), Thailand (150,000 cases, 5 deaths) and Malaysia (111,400 cases, 5 deaths) (Bouzidi et al., 2026). While there is clear evidence of a significant rise in the frequency of dengue cases, the patterns observed in these data are more likely linked to climate change, urbanization, the spread of Aedes vectors, increased surveillance efforts, and greater human mobility than to changes in serotype virulence.

In global epidemiology, the periodicity of the two predominant serotypes, DENV-1 and DENV-2, is constant, but their stable dominance is not. Epidemiological surveillance has shown that serotype replacement often occurs before large outbreaks, implying a significant role for viral evolution and declining population immunity as major factors in epidemic emergence. For instance, Singapore experienced a significant outbreak of DENV-1 infection in 2013–14 following the replacement of the dominant DENV-2 lineage, while replacement of DENV-2 lineages was linked to changes in the outbreak pattern in Guangzhou, China (Ma et al., 2021; Jiang et al., 2022). In the same way, DENV-1 was the most common serotype detected in laboratory-confirmed dengue cases in Arunachal Pradesh, India, in 2015 (66/73 cases), followed by DENV-2 and mixed infections (83). These observations suggest that genotype (not necessarily serotype) variability is a key factor in shaping outbreak dynamics.

There is significant geographical and temporal variation and inequality in the epidemiology of dengue across the six WHO Regions, including the burden of disease, the presence of serotypes, the dynamics of dengue genotypes, surveillance activities, and responses to outbreaks. Dengue is highly endemic in South-East Asia and has had significant recent spread in Indonesia, India, Bangladesh, Thailand, Malaysia, and Viet Nam, with DENV-1 and DENV-2 often predominant in some settings, and changes in the serotypes circulating and in monsoon-associated transmission likely contributing to recurrent outbreaks (Bouzidi et al., 2026; Anwar et al., 2020; Anwar et al., 2022a). Recurrent outbreaks and lineage replacement have been reported in the Western Pacific region, including the DENV-1 outbreak in Singapore in 2013–2014 and genotype changes accompanying the introduction of dengue transmission in Guangzhou, China (Ma et al., 2021; Jiang et al., 2022). The Americas are the region with the highest reported burden, with over 12.6 million dengue cases reported across Latin America in 2024 and approximately 7700 deaths reported in the region, with DENV-1 the predominant strain in several countries such as Brazil, Colombia, and Mexico (*Record Dengue Outbreak in Americas Kills 7,700 this Year, PAHO Says*, 2025). Recurrent outbreaks have been reported in the Eastern Mediterranean Region, including in Pakistan in 2011, with 21,685 confirmed DENV-2 cases and 350 deaths reported during the outbreak in Lahore (Anwar et al., 2022b; Rehman et al., 2022). The African Region is becoming more affected, but undercharacterized, as the burden of disease may be underestimated due to limited laboratory capacity, inconsistent surveillance, and lack of diagnostic and vector-control resources; DENV-2, DENV-3, and DENV-1 have been reported to be circulating, and major outbreaks have been reported in countries in the Region such as Burkina Faso, Cabo Verde, and Mali. Europe is not an endemic region where dengue was traditionally recorded, but rather a new potential transmission area with locally acquired dengue cases reported in France, Italy, Spain, Croatia and Portugal, where vector suitability is likely to be rising with warmer temperatures, more rainfall and growing Aedes populations, which may lead to seasonal transmission (Rovida et al., 2024). Variations in case definitions, laboratory confirmation, intensity of surveillance, and reporting completeness across the regions make direct comparisons of reported case numbers challenging, and the variations in the serotypes and lineages of viruses circulating, as well as changes in population immunity, urbanization, population movement, and vector expansion, affect the magnitude of outbreaks and geographical spread. Therefore, simultaneous laboratory, epidemiological, genomic, and entomological surveillance, along with prompt vector control, clinical preparedness, and coordinated public-health responses, are critical for early detection and effective management of the changing dynamics of dengue transmission.

In summary, the available evidence suggests a complex epidemiological picture with a dynamic interplay among serotype replacement, genotype evolution, host immunity, and environmental changes that affect global dengue transmission. Therefore, integrated genomic surveillance, climate-based forecasting, vector monitoring, and international surveillance systems with common standards are required to predict future outbreaks and cannot replace serotype distribution (*PAHO Warns of Increased Risk of Dengue Outbreaks Due to Circulation of DENV-3 in the Americas*, 2025; Kothari et al., 2025; World Health Organization, 2026; Shete et al., 2026; Kalimuddin et al., 2025).

## 7 Control strategies: current evidence and remaining challenges

Although there has been significant progress in dengue prevention, sustained control has not yet been achieved in all dengue-endemic areas, underscoring that dengue is a multifactorial problem. The WHO identified the main factors causing rapid dengue outbreaks to be rapid urbanization, growing population density, poor water management, and the spread of Aedes mosquito breeding grounds (Bouzidi et al., 2026). Thus, priority is given to an integrated approach to control, personal protection, surveillance, vaccination, and community participation rather than to any single measure.

One of these interventions is surveillance, which is the backbone of Dengue Fever control; when performed at an early stage, it facilitates early prediction of outbreaks and vector control actions (Naderian et al., 2025). But effectiveness depends on the quality of surveillance. In the case of the 2005 Singapore outbreak, limitations in surveillance and an unsustainable level of vector control contributed to the expansion of the epidemic, as analyses suggested (Chermahini et al., 2025; Mahooti et al., 2026). Based on these observations, surveillance systems need to be complemented by prompt public health action to effectively reduce transmission.

Vector-control programs have also been expanded to include more than just the traditional insecticide programs. New strategies and techniques for controlling vectors should be identified and differentiated by mechanism and implementation. The genetically modified mosquito strategies, SIT, and IIT are different biological or genetic approaches, whereas IGRs mainly target mosquito immature stages. The interventions vary significantly in evidence, operational requirements, costs, scalability, and sustainability. However, long-term efficacy, ecological impact, public acceptance, and cost-effectiveness have not been fully documented, which is a barrier to their widespread use. The majority of the available evidence comes from small-scale field study evaluations; however, large-scale comparative studies demonstrating continued epidemiological benefit are limited (Abidemi and Aziz, 2020; Utarini et al., 2021; Yang et al., 2025; Pang et al., 2017; Pourzangiabadi et al., 2025).

Vaccination is a supplementary measure of protection, and not all populations can be vaccinated. The response to vaccines also highlights the need for serotype balance and the immune background level for dengue prevention. A 2025 systematic review of dengue vaccines in adolescents showed that TAK-003 achieved high seropositivity across all four DENV serotypes, elicited a strong response to DENV-2, and sustained antibody levels for up to 3 years. Dengvaxia-induced neutralizing antibodies, on the other hand, and clinical protection were more desirable in those who were already protected against dengue. Adverse events were mild to moderate for most vaccine types, and serious events occurred in a small proportion of vaccinees overall. Caution is warranted with immune enhancement in seronegative populations, and the different serotype-specific efficacies should all be taken into account. The results of these studies suggest that vaccine efficacy should be measured in terms of serotype-specific efficacy, longevity of the neutralizing antibody response, baseline serostatus, and safety, and not efficacy alone. Dengvaxia® (CYD-TDV) is a live-attenuated tetravalent dengue vaccine whose use is restricted to individuals with evidence of previous dengue infection and within the age groups and conditions specified by the relevant national regulatory authorities. WHO recommendations should be distinguished from national regulatory approvals, as vaccine licensure, eligible age groups, serostatus requirements, and programmatic use may differ between countries.TAK-003 (Qdenga®) is a live-attenuated tetravalent dengue vaccine containing components representing DENV-1, DENV-2, DENV-3 and DENV-4; it uses an attenuated DENV-2 strain as the genetic backbone. Its efficacy varies according to infecting serotype, baseline serostatus, age, and prior dengue exposure. Vaccine effectiveness, however, depends on baseline serostatus (not all catch-up vaccines are effective), circulating serotype, age, and prior exposure to dengue, suggesting that vaccination should be used in addition to, rather than in lieu of, surveillance and vector-control programs, as shown in Table 6.

**Table 6:** Evidence-based comparison of dengue control and prevention strategies.

Intervention	Effectiveness / evidence	Key limitations	Cost / resource requirement	Scalability	Sustainability	Implementation setting	Critical interpretation	References
Community-based source reduction	Removal of standing water and breeding containers can reduce *Aedes* breeding sites and household vector density when consistently implemented.	Requires sustained community participation; behavioural adherence may decline over time.	Low–moderate; mainly requires personnel, education and community mobilization.	High where community and municipal systems are functional.	Moderate–high when incorporated into routine community programmes.	Endemic urban, peri-urban and community settings.	Essential foundation of integrated vector management but insufficient as a stand-alone strategy during intense transmission.	(Bouzidi et al., 2026; Abidemi and Aziz, 2020; Utarini et al., 2021; Yang et al., 2025; Pang et al., 2017; Pourzangiabadi et al., 2025)
Chemical/insecticide-based vector control	Adulticiding and insecticide-treated interventions can rapidly reduce adult mosquito populations and provide short-term suppression.	Insecticide resistance, environmental concerns and rebound of vector populations can reduce sustained effectiveness.	Moderate–high, depending on chemicals, equipment and repeated application.	High for emergency outbreak response where operational capacity exists.	Moderate–low if heavily dependent on repeated chemical application.	Outbreak response and endemic areas with established vector-control infrastructure.	Useful for rapid suppression but should be combined with source reduction and resistance monitoring.	(Bouzidi et al., 2026; Abidemi and Aziz, 2020; Utarini et al., 2021; Yang et al., 2025; Pang et al., 2017; Pourzangiabadi et al., 2025)
Larval control / insect growth regulators (IGRs)	IGRs such as diflubenzuron and methoprene interfere with immature mosquito development and can reduce larval density.	Limited effect on adult mosquitoes; effectiveness depends on reaching breeding habitats and may be affected by resistance.	Low–moderate; requires repeated treatment and larval surveillance.	Moderate–high in identifiable breeding sites.	Moderate when integrated with environmental management.	Urban/peri-urban areas with identifiable and treatable breeding sites.	Particularly useful as a complementary larval intervention rather than an independent control strategy.	(Abidemi and Aziz, 2020; Utarini et al., 2021; Yang et al., 2025; Pang et al., 2017; Pourzangiabadi et al., 2025)
Wolbachia-based population replacement	Wolbachia-infected mosquitoes reduce vector competence for dengue; field programmes have demonstrated promising reductions in mosquito populations.	Requires establishment and maintenance of Wolbachia in local mosquito populations; ecological and operational performance may vary geographically.	Moderate–high initial programme investment with potential long-term reduction in recurrent intervention needs.	Potentially high, but requires local infrastructure, monitoring and regulatory approval.	Potentially high after stable Wolbachia establishment.	Suitable endemic urban/peri-urban settings with strong programme infrastructure.	Should be distinguished from GM mosquitoes, SIT and IIT; it is a biological population-replacement approach.	(Abidemi and Aziz, 2020; Utarini et al., 2021; Yang et al., 2025; Pang et al., 2017; Pourzangiabadi et al., 2025)
Genetically modified mosquitoes	Genetic modification can reduce vector populations or alter vector competence; field programmes have demonstrated promising reductions in mosquito populations.	Long-term epidemiological effectiveness, ecological effects, public acceptance and regulatory requirements require continued evaluation.	High initial technical and regulatory requirements.	Potentially high, but dependent on regulatory approval, mass-rearing and release infrastructure.	Uncertain; repeated releases may be required depending on the technology.	Selected endemic settings with regulatory and technical capacity.	Promising technology but should not be equated with Wolbachia or sterile-male approaches.	(Fraser Jr., 2012; Favia, 2015; Rather et al., 2017)
Sterile Insect Technique (SIT)	Release of sterile males can reduce reproductive success and suppress mosquito populations when releases are sufficiently frequent and spatially coordinated.	Requires continuous mass production and release; effectiveness depends on release quality, coverage and ecological conditions.	High operational requirement because of mass rearing, sterilization and repeated releases.	Moderate–high where mass-rearing and release infrastructure is available.	Moderate; sustained releases may be required.	Targeted endemic urban/peri-urban areas and integrated vector-control programmes.	Population-suppression technology that differs mechanistically from Wolbachia population replacement and GM mosquitoes.	(Abidemi and Aziz, 2020; Utarini et al., 2021; Yang et al., 2025; Pang et al., 2017; Pourzangiabadi et al., 2025)
Incompatible Insect Technique (IIT)	Uses *Wolbachia*-induced cytoplasmic incompatibility to reduce viable offspring following release of incompatible males.	Requires reliable sex separation, mass rearing and maintenance of appropriate Wolbachia infection status.	High initial and operational requirements.	Moderate, depending on production and release capacity.	Moderate, with repeated releases generally required for suppression.	Targeted endemic areas with adequate mosquito-production and monitoring infrastructure.	Should be considered a distinct population-suppression strategy and may be combined with SIT or other vector-control approaches.	(Abidemi and Aziz, 2020; Utarini et al., 2021; Yang et al., 2025; Pang et al., 2017; Pourzangiabadi et al., 2025)
Vaccination	Dengue vaccines can reduce symptomatic and severe dengue in eligible populations, but efficacy varies according to vaccine, serotype, age and baseline serostatus.	Serotype-specific differences, baseline serostatus, age restrictions and variable protection limit universal application.	Moderate–high, including vaccine procurement, delivery and surveillance.	Moderate–high where immunization systems are established and eligible populations can be identified.	Moderate, depending on duration of protection and need for subsequent doses.	Endemic settings implementing evidence-based national vaccination programmes.	Vaccination is complementary to, rather than a replacement for, vector control and surveillance.	(Bouzidi et al., 2026; Wilder-Smith, 2024; Aguiar et al., 2016; Izmirly et al., 2020)
Clinical surveillance and case management	Early recognition of warning signs, appropriate fluid management and monitoring can reduce progression to severe dengue and mortality.	Requires trained healthcare personnel, diagnostic capacity and adequate clinical infrastructure.	Moderate–high, particularly during outbreaks requiring additional healthcare capacity.	High within functioning health systems.	High when incorporated into routine clinical services.	Primary, secondary and tertiary healthcare settings.	Essential for reducing mortality but does not directly interrupt mosquito transmission.	(Bouzidi et al., 2026; Yang et al., 2025)
Genomic surveillance	Sequencing can identify serotype/genotype circulation, lineage replacement and introduction of emerging variants, supporting epidemiological interpretation.	Requires laboratory infrastructure, sequencing capacity, bioinformatics expertise and representative sampling.	Moderate–high depending on sequencing platform and laboratory capacity.	Moderate, with greatest feasibility through regional/reference laboratories.	High if integrated into national surveillance networks and data-sharing systems.	National reference laboratories, research centres and outbreak investigations.	Provides information that conventional case surveillance cannot capture and is particularly valuable for monitoring lineage replacement.	(Ma et al., 2021; Jiang et al., 2022; *Record Dengue Outbreak in Americas Kills 7,700 this Year, PAHO Says*, 2025; *PAHO Warns of Increased Risk of Dengue Outbreaks Due to Circulation of DENV-3 in the Americas*, 2025; Kothari et al., 2025; World Health Organization, 2026; Shete et al., 2026; Kalimuddin et al., 2025)
Early-warning and climate-informed surveillance	Integration of epidemiological, vector, climatic and environmental indicators can support earlier identification of increasing transmission risk.	Prediction accuracy varies with data quality, local ecology and model assumptions; forecasts require timely public-health action.	Moderate, depending on data systems and modeling capacity.	High where digital surveillance and meteorological data are available.	High when integrated into routine surveillance.	National/regional surveillance systems and outbreak-prone endemic areas.	Early warning is most useful when linked to predefined response thresholds and rapid vector-control deployment.	(*PAHO Warns of Increased Risk of Dengue Outbreaks Due to Circulation of DENV-3 in the Americas*, 2025; Kothari et al., 2025; World Health Organization, 2026; Shete et al., 2026; Kalimuddin et al., 2025)
Community participation and health education	Education, household source reduction and behavioural interventions can reduce breeding opportunities and improve adherence to control measures.	Behavioural change is difficult to maintain; effectiveness depends on trust, communication and continuous engagement.	Low–moderate relative to technological interventions.	High with strong local-government and community networks.	Moderate–high when institutionalized within routine public-health programmes.	Household, school, workplace and community settings.	A cross-cutting component that strengthens the effectiveness and sustainability of other interventions.	(Bouzidi et al., 2026; Abidemi and Aziz, 2020; Utarini et al., 2021; Yang et al., 2025; Pang et al., 2017; Pourzangiabadi et al., 2025)

Overall, the available evidence supports integrated dengue management rather than reliance on a single intervention, although the relative contribution and effectiveness of individual components vary across epidemiological, ecological, and health-system contexts. High-quality surveillance, targeted vaccination, evidence-based vector control, environmental sanitation, and community engagement must be coordinated to ensure sustainable control. The standardized assessment of novel vector-control technologies, long-term vaccine protection against various serotypes, and integrated control strategies with cost-effective frameworks applicable across a variety of epidemiological contexts should be emphasized.

Clinical surveillance and case management are complementary components of dengue control because they primarily reduce severe outcomes rather than directly interrupt transmission. Early recognition of warning signs, appropriate fluid management, monitoring of hemodynamic status and organ function, and timely referral of patients with severe dengue are essential for reducing mortality. These measures should therefore be evaluated separately from vector-control interventions when assessing the effectiveness of dengue control.

In general, when interventions are applied as an integrated, context-specific approach, dengue control is most effective, not as individual technologies. Community-based source reduction and larval and insecticide-based control are essential and share common traits, while Wolbachia, genetically modified mosquitoes, SIT and IIT are all biological or genetic vector-control technologies with unique mechanisms, requirements and evidence. Vaccine use offers another level of protection, but factors of vaccine efficacy, age, baseline serostatus, and circulating serotypes need to be taken into account. The main outcomes of clinical surveillance and case management are to reduce morbidity and mortality, while genomic surveillance and early-warning systems will help identify changes in serotype or lineage and forecast increased transmission risk. The comparative effectiveness, cost, scalability and sustainability of these interventions are dependent on vector ecology, capacity of the health system, regulatory frameworks and community acceptance and should be tailored to local epidemiological and operational conditions for integrated programs.

## 8 Challenge

Although there have been considerable advances in surveillance, vaccination and vector control, effective control of dengue still faces many difficulties arising from the interlinked nature of the scientific, clinical, epidemiological and public health challenges that underpin the disease. Laboratory and diagnostic capacity are limited, surveillance systems are fragmented, healthcare infrastructure and staffing are inadequate, insecticide resistance is a challenge, and limited community engagement, especially in LMICs, is a key barrier (*Dengue- Global Situation*, 2025). The challenges in endemic areas are diverse, and therefore there is a need to improve health systems, strengthen biomedical and public health interventions, and develop them.

A significant hurdle to preventing dengue is that existing vaccines do not provide complete protection across all populations. Dengvaxia® has been licensed in a number of dengue-endemic countries, but is recommended for use in individuals with evidence of prior dengue infection, as vaccinating seronegative individuals may increase the risk of severe dengue after a subsequent natural infection (*DENGVAXIA*, 2025; *Dengue- Global Situation*, 2025; Aguiar et al., 2016). These restrictions mean that vaccine efficacy is influenced by vaccine properties and by factors such as baseline immune status, circulating serotypes, age, prior exposure, and local epidemiology, making universal vaccine strategies impractical.

The dynamic nature of DENV-1 and DENV-2 circulation and genetic evolution is another challenge that makes it difficult to predict outbreaks and to estimate in the long-term the performance of potential vaccines. The Guangzhou and Singapore outbreaks illustrate how outbreaks can shift quickly after the introduction of a new genotype or lineage, even without the advent of a new serotype (World Health Organization, 2026). Likewise, mutations in the DENV-2 envelope (E) protein could impact antigenicity and antibody-mediated neutralization, which might impact vaccine-mediated protection against emerging variants (Lim et al., 2019). Given that direct clinical evidence of an association between DENV-2 mutations and vaccine failure remains limited, and many current conclusions are based on molecular and experimental studies, it remains unclear whether DENV-2 mutations are directly linked to vaccine failure.

Antibody-dependent enhancement (ADE) is a significant biological factor to consider when understanding the pathogenesis of Dengue virus infection, designing a Dengue vaccine, and managing the disease. In some secondary infections, subneutralising or non-neutralizing antibodies produced during the primary infection can enable Fc receptor-mediated viral entry into susceptible immune cells, which may lead to enhanced viral replication and raise the likelihood of severe dengue infection such as dengue hemorrhagic fever (DHF) and dengue shock syndrome (DSS) (Lim et al., 2019; Izmirly et al., 2020). However, severe disease is not invariably seen in secondary infection with dengue, and clinical outcomes will depend on several factors such as concentration and specificity of antibody, infecting serotype and genotype, interval between the infection, age of host, immune history, and other host-related factors. Moreover, cross-reactive immune responses among flaviviruses, such as DENV and Zika virus, complicate differential diagnosis and vaccine development. Therefore the concentration, specificity, and immunological context of the antibodies in the body can be a source of protection or cause improved infection.

In general, the problem of reduced dengue control effectiveness is not caused by a single intervention or biological factor, but by the interplay among viral evolution, complex host immunity, diagnostic and surveillance limitations, vector ecology, insecticide resistance, health-system constraints, and socioeconomic and environmental factors. Advances in vaccine development to offer broad and long-lasting protection, region-specific and integrated vector-control strategies, enhanced surveillance of genomics and epidemiology, better diagnostic tools, and longitudinal immunological studies should therefore be the focus of future research. Access to climate-informed early-warning systems, sustainable community engagement and equitable access to vaccination, prevention, diagnosis and adequate clinical management, especially in resource-limited areas, is equally important.

## 9 Future perspectives

One constraint of existing dengue studies is not a lack of mechanistic evidence, but rather that the evidence is fragmented. Most investigations focus on viral evolution, immunopathology, molecular epidemiology, or clinical outcomes, and do not provide a comprehensive understanding of serotype-specific pathogenicity (Martínez et al., 2024; Giovanetti et al., 2022; Bouzidi et al., 2026; Alagarasu et al., 2021; Ravi et al., 2025; Anwar et al., 2020; Anwar et al., 2022a; Anwar et al., 2022b; Rehman et al., 2022; Ma et al., 2021; Jiang et al., 2022; *Record Dengue Outbreak in Americas Kills 7,700 this Year, PAHO Says*, 2025; *PAHO Warns of Increased Risk of Dengue Outbreaks Due to Circulation of DENV-3 in the Americas*, 2025). Thus, the higher severity often reported with DENV-2 may be due to viral characteristics or to differences in host immunity, population structure, and epidemiology that are not yet known (Torres et al., 2021; Martina et al., 2009; Balmaseda et al., 2006).

Yet another is the dependence on experimental models. The evidence for improved viral fitness, immune escape, endothelial dysfunction due to NS1, as well as cytokine dysregulation comes mainly from *in vitro* systems or animal studies, with few prospective studies in humans (Chutia et al., 2026; Sinha et al., 2024; Chen et al., 2018; Bhatt et al., 2020; Pang et al., 2007; Halstead, 2019; Guabiraba and Ryffel, 2014; Shrivastava et al., 2020). This difference makes it difficult to translate mechanistic observations into clinical markers or therapeutic targets and contributes to discrepancies between experimental and outbreak studies (Chutia et al., 2026; Sinha et al., 2024; Gupta et al., 2021).

Likewise, molecular epidemiological studies have greatly advanced knowledge of genotype diversity and lineage replacement, but such studies are largely descriptive (Martínez et al., 2024; Giovanetti et al., 2022; Ma et al., 2021; Jiang et al., 2022). While the replacement of the genotype is often a precursor to major epidemics, there is limited evidence so far to support direct causal links between viral genetic variation and disease severity, due to the fact that often, host immune history, antibody-dependent enhancement, vector competence, and environmental pressures are not analysed together (Martina et al., 2009; Sangkawibha et al., 1984; Rico-Hesse et al., 1997; Guzman and Kouri, 2003; Burke et al., 1988; Ma et al., 2021; Jiang et al., 2022).

Another challenge is that knowledge of dengue pathogenesis remains incomplete, limiting therapeutic development. Despite the fact that severe dengue is the product of an integrated response of inter-related processes of virus replication, endothelial damage, metabolic reprogramming, and immune dysregulation, most investigational strategies focus on targeting individual viral proteins or inflammatory pathways (Chutia et al., 2026; Sinha et al., 2024; Chen et al., 2018; Bhatt et al., 2020; Pang et al., 2007; Halstead, 2019; Guabiraba and Ryffel, 2014; Shrivastava et al., 2020).

Lastly, a balance between serotype-specific vaccine protection and efficacy alone needs to be emphasized in future vaccine development. Long-term protection will require vaccines that generate broadly and persistently neutralizing immune responses without causing immunopathology, given the potential for antibody-dependent enhancement and the importance of variability in vaccine effectiveness across serotypes and baseline seropositivity (*DENGVAXIA*, 2025; Wilder-Smith, 2024; *Dengue- Global Situation*, 2025; Aguiar et al., 2016). Together, these challenges emphasize the need for the next step to be the development of a unifying framework for precision dengue prevention and management, one that combines molecular virology, systems immunology, clinical epidemiology, and real-time genomic surveillance.

In the end, the dengue virus is a vector-borne virus that poses an immense global health threat, with about 3 billion people living in regions susceptible to its spread. The dengue virus has four serotypes, but this article focuses on DENV-1 and DENV-2, which cause a broad spectrum of disease, ranging from mild signs to critical health emergencies. It has been reported to have a higher mortality rate across various geographic regions. The clinical symptoms of DENV-1 and DENV-2 infections vary due to distinct interactions among viral components, host immune responses, and vector distribution, ultimately resulting in fever, hemorrhage, and shock. The severity of a disease is a function of viral load, viral genotype, infection sequence, previous dengue exposure, host immunity, age, and geographical and epidemiological factors. Dengue fever is a significant global health burden, with 100–400 million cases annually, high economic costs, substantial social impact, and significant health care needs. The reasons for the continued rise in dengue fever cases are urbanization, climate change, and ineffective vector control programs.

Several strategies, including vector control, vaccination, early detection, and adequate treatment, are needed to control the spread of dengue fever. The Dengvaxia vaccine has proven effective in reducing the incidence of severe dengue, among other benefits. When integrated vector management strategies are coupled with mosquito monitoring and management programs, disease transmission is more controllable. Strict control measures are necessary to address dengue fever (DF) caused by DENV-1 and DENV-2, which can have considerable adverse health effects on the population. The coordinated roll-out of vaccines, vector surveillance, and prompt diagnosis of the disease have helped reduce the disease burden of Dengue Fever in countries.

## 10 Conclusion

Dengue continues to be a significant public health problem worldwide, with significant transmission in tropical and subtropical regions and higher risk of transmission in emerging transmission areas. This review emphasized DENV-1 and DENV-2 specifically and revealed that both serotypes have common major pathogenic mechanisms such as viral replication, endothelial dysfunction associated with NS1, inflammatory mechanisms, metabolic changes, and ADME, and that clinical outcomes are significantly different between populations and epidemiological contexts. There is currently no evidence of a fixed ranking of infection severity for DENV-1 or DENV-2. In contrast, the severity of disease seems to depend on the interaction of viral genotype and lineage, viral load, sequence of infection, previous exposure to dengue virus, immune status, age, geographical location, and other epidemiological and environmental factors (Yung et al., 2015; Chutia et al., 2026; Sinha et al., 2024; Sangkawibha et al., 1984; Rico-Hesse et al., 1997; Guzman and Kouri, 2003; Burke et al., 1988; Fried et al., 2010; Nisalak et al., 2003; Yacoub and E. M. W. W. W. W, 2017; Chinnawirotpisan et al., 2008; Ler et al., 2011; Balmaseda et al., 2006; Guzmán et al., 2000; Gupta et al., 2021).

There are key questions to be addressed regarding the role of direct genetic variation in the virus in clinical severity. Direct comparison between the two serotypes is limited and severe dengue is a feature of DENV-2 in several epidemiological and experimental studies, but it has also been observed during outbreaks dominated by DENV-1 (Sangkawibha et al., 1984; Rico-Hesse et al., 1997; Guzman and Kouri, 2003; Burke et al., 1988; Fried et al., 2010; Nisalak et al., 2003; Yacoub and E. M. W. W. W. W, 2017; Chinnawirotpisan et al., 2008; Ler et al., 2011; Balmaseda et al., 2006; Guzmán et al., 2000; Gupta et al., 2021). In this context, therefore, observations of serotype-associated severity must be understood as context-dependent associations as opposed to evidence of intrinsic and universal differences in virulence. Differences in disease outcomes must be viewed in the context of genotype and lineage replacement, host immune history, antibody responses, vector ecology, and geographical and population-level factors.

The present preventive and control methods are of great value but also have significant limitations. The efficacy of vaccinations depends on the vaccine's properties, age, serostatus, prior exposure to dengue virus, circulating serotype, and the operational conditions, cost, and community engagement of vector-control measures, which may also be affected by the development of insecticide resistance. Next-generation tools such as Wolbachia-based approaches, genetically modified mosquitoes, SIT, and IIT show promise but have yet to be assessed for long-term epidemiological efficacy, scalability, sustainability, safety, and cost-effectiveness. This means that there is no single intervention to be implemented across all transmission settings, and that integrated, locally adapted strategies continue to be needed.

Standardized prospective studies with multicentre collection that combine viral genomics, quantitative viral load measurements, longitudinal immunological profiling, and harmonized clinical severity classification should be prioritized for future studies. Enhancements in diagnostic capacity, serotype and genomic surveillance, climate-based early warning systems, and real-time epidemiological monitoring will be critical for detecting shifts in transmission and viral circulation. Other research needs include assessing the durability and safety of vaccines, comparative effectiveness and cost-effectiveness of vector-control technologies, and equitable access to prevention and clinical care. This will help differentiate intrinsic virally mediated determinants from host-, ecological-, and context-dependent determinants of dengue severity and enhance precision in dengue prevention, surveillance, and clinical management.

## CRediT authorship contribution statement

**Shairy Priya:** Writing – original draft. **Rishabha Malviya:** Writing – review & editing, Supervision. **Phool Chandra:** Writing – original draft. **Sathvik Belagodu Sridhar:** Writing – original draft. **Javedh Shareef:** Methodology, Data curation. **Tarun Wadhwa:** Project administration, Conceptualization. **Daniel Arockiam:** Writing – review & editing, Funding acquisition.

## Clinical trial number

Not applicable.

## Consent to publication

Not applicable.

## Ethics approval and consent to participate

Not Applicable.

## Funding

No funding received for the study.

## Declaration of competing interest

The Authors have no conflicts of interest.

## Data Availability

Not applicable.
